# Hypoxia imaging in cells and tumor tissues using a highly selective fluorescent nitroreductase probe

**DOI:** 10.1038/s41598-017-09525-2

**Published:** 2017-08-23

**Authors:** Dan Yang, Hang Yu Tian, Tie Nan Zang, Ming Li, Ying Zhou, Jun Feng Zhang

**Affiliations:** 1 College of Chemical Science and Engineering, Yunnan University, Kunming, 650091 PR China; 2 Institute of Life Sciences, Yunnan University, Kunming, 650000 China; 3 College of Chemistry and Chemical Engineering, Yunnan Normal University, Kunming, 650500 PR China

## Abstract

Hypoxia is a characteristic of locally advanced solid tumors, resulting from an imbalance between oxygen consumption and supply. In hypoxic solid tumors, an increased expression of nitroreductase (NTR) is detected, therefore, the development of NTR-targeted fluorescent probes to selectively and efficiently detect hypoxia *in vivo* is of utmost importance. In this study, a probe (1) has been designed and tested for effective optical detection of NTR *in vitro* and *in vivo*. The reduction of probe (1), catalyzed by NTR, resulted in changes of the electron-withdrawn nitrogen group into an electron-donation amino group. In addition, breakage of the O-C bond ensured selective fluorescence enhancement. The *in vitro* response towards exogenous NTR, from rat liver microsomes, resulted in the optical enhancement during the detection process. *In vivo* imaging of caerorhabditis elegans (*C.elegan*) further confirmed the detection of NTR by probe (1). Moreover, probe (1) was successfully used for the detection of hypoxia in both HI5 cells, and a murine tumor model, which demonstrates the potential of probe (1) for application in fluorescence bioimaging studies, and tumor hypoxia diagnosis.

## Introduction

Nitroreductases (NTRs) are a family of flavin-containing enzymes that catalyze the reduction of nitroaromatic compounds to the corresponding amines using either NADH or NADPH as a source of reducing equivalents^1, 2^. NTRs play important roles in the bioremediation and degradation of toxicity of organic nitrogen compounds mediated by bacteria^3^. Under hypoxic conditions, overexpression of intracellular reductases, including NTRs, DT-diaphorase, and azoreductase was observed^4^. In solid tumors, expression of NTRs is directlyrelated to the hypoxic status. Therefore, our goal was to measure hypoxia in tumor cells or tumor-derived tissue^5–7^.

Fluorescence imaging is a promising approach for monitoring bioactive molecules in living systems^8–11^. Fluorescence imaging is a sensitive, selective, rapid, and an efficient bioanalytical tool for tracing the state, changes, and activities of targets *in vivo*, thereby facilitating progress in the fields of cell biology,and imaging using therapeutics^12–16^. Recently, the development of sensors that detect NTRs have attracted much attention because of theirpotentialto detect the hypoxic status of a tumor^17–20^. In these newly generated fluorescent probes, a selective ‘switch’ mechanism is used. The large electronic change resulting from the conversion of the electron-withdrawn nitro group to the electron-donation amino group leads to breakage of the O (or N)-C bond, which ensures recovery of the fluorescence signal and an ‘off-on’ recognition strategy. In addition, this reaction-based sensing mechanism guarantees that the probes have high selectivity towards NTRs. However, *in vivo* applications of NTR probes for selective imaging,and therapy of intracellular hypoxia are challenging. Given its promising properties, nitrobenzyl redox switching for NTR active materials was chosen as a preferred detection signal for the design of our NTR-switchable probe. We hypothesized that the probe could monitor intracellular hypoxic levels, and be used to investigate the relation between the hypoxic status and NTR expression level in cells, C. *elegans* and tumor tissues.

We fabricated a novelprobe, composed of a typical donor-π-acceptor featured chromophore, dicyanomethylene-4H-pyran (DCM), and a nitrobenzene group, to sensitively monitor the NTR level. The elongated conjugated system and asymmetric structure result in a push-pull construction that allows the designed sensor to have a stronger fluorescence signal, and shorter emission wavelength in the presence of NTR when compared tocontrol conditions in which NTR is not present. In this article, we present the synthesis and optical properties of a novel NTR sensitive probe (1). We demonstratedthat probe (1) could selectively and accurately measure endogenous and exogenous NTR levels from different sources with a unique fluorescence enhancement and a large blue-shift. Thus, probe (1) maybe a promisingtool for monitoring intracellular hypoxic levels, and maybe used for measuringthe hypoxic state in tumors.

## Results and Discussion

### Synthesis of probe (1)

**Figure 1:**
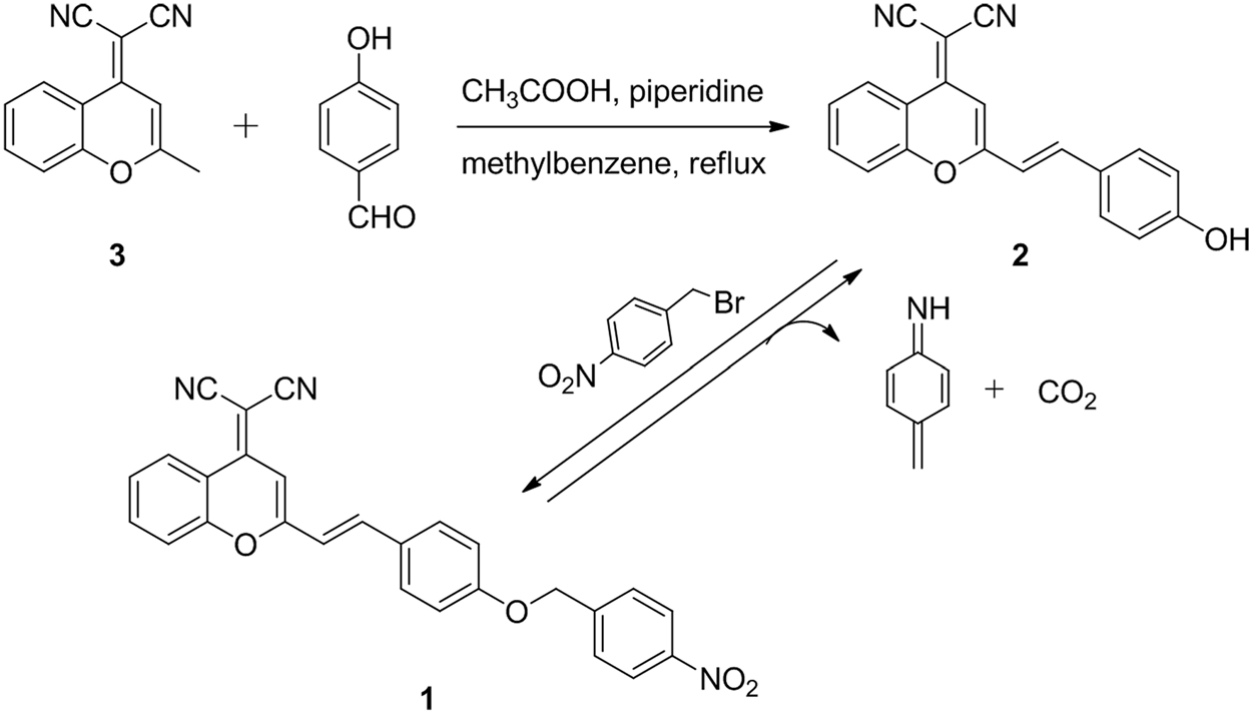
Synthetic route of probe (1), and the reactivity of probe (1) with nitroreductase.

Compound 3, synthesized as previously described^21^, reacted with 4-hydroxybenzaldehyde to generate intermediate compound 2^22^. Subsequently, intermediate 2 underwent a condensation reaction under relatively mild conditions to form probe (1) (Fig. 1). Synthetic details and characterization of the newly derived probe (1) can be found in the ESI.

### Fluorescence responses of probe (1) with NTR

**Figure 2:**
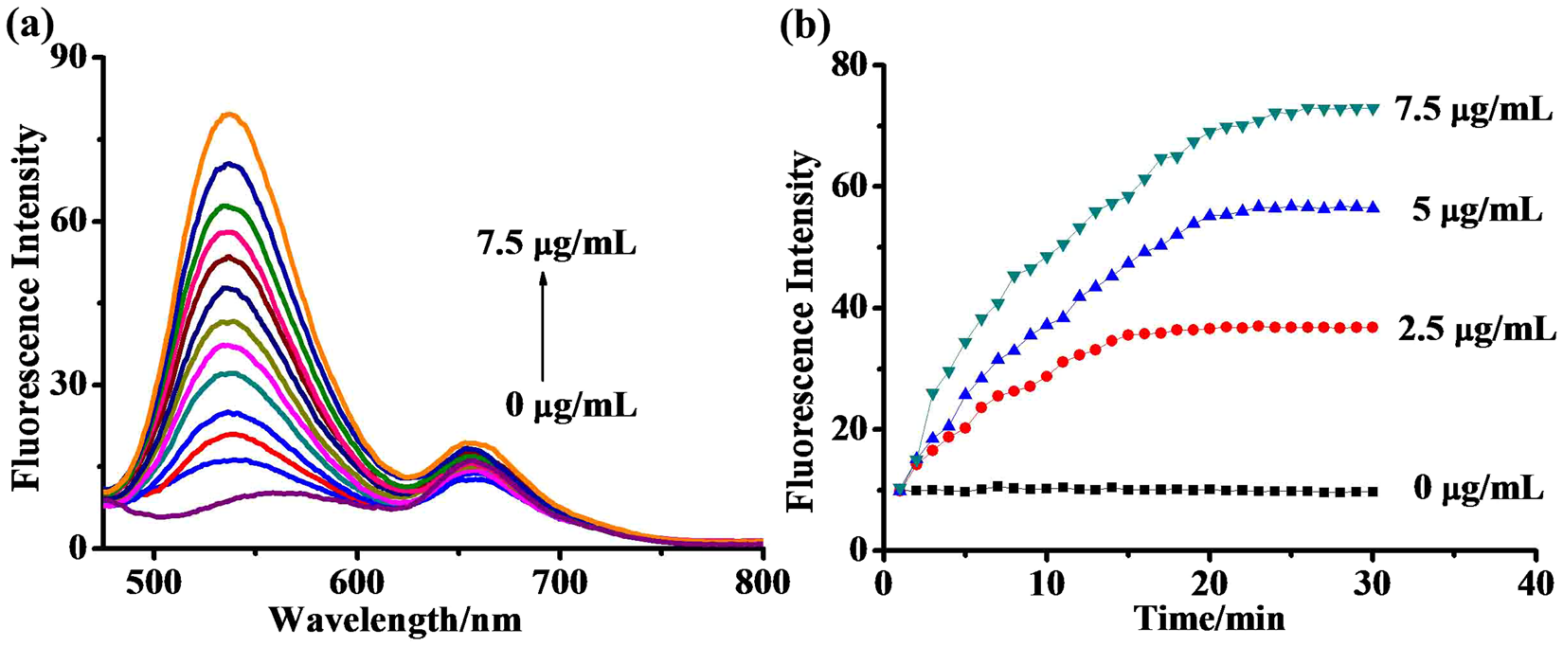
(**a**) Fluorescence responses of probe (1) (1.0 × 10^−5^ M) to different concentrations of nitroreductase (NTR) in PBS buffer with 1% DMSO and 10 μM NADH. (**b**) A plot showing the fluorescence intensityof probe (1) (1.0 × 10^−5^ M) at 537 nm vs. the reaction time in the presence of different concentration of NTR: 0 (control), 2.5, 5, and 7.5 µg/mL. Measurements were performed in 10 mM PBS buffer at 37 °C (pH = 7.4).

To set up the experimental conditions, we compared the effect of the pH on the emission of 1 versus emission spectra of the reaction of probe (1) catalyzed by NTR. (Fig. S2). Because probe (1) was stable and reduced well by NTR under physiological conditions, pH and temperature conditions were chosen as pH 7.4 and 37 °C. The absorption andemission spectra of probe (1) (2.0 × 10^−5^ M) in the presence and absence of NTR were evaluated in a DMSO/PBS buffer (0.01 M, pH 7.4) (1:99 v/v). Probe (1) exhibited broad absorption in the range of 360–520 nm. The reduction of probe (1) catalyzed by NTR resulted in a decrease of the absorption peak at 345 nm (Fig. S3, ESI). The fluorescent probe itself showed a very weak emission at 660 nm, and the addition of NTR and NADH to the solution of 1,led to an 11-fold increase in fluorescence enhancement at a wavelength of 537 nm. The fluorescence responses of probe (1) to NTR at different concentrations are shown in Fig. 1a. Increasing NTR concentrations showed a gradual increase in fluorescence intensity at 537 nm, the lowest detectable concentration of NTR being 24.5 ng/mL (Fig. S5). The kinetic curves of the fluorescence intensities of probe (1) reacting with different concentrations of NTR resulted in a plateau level (Fig. 2b). The plateau was reached roughly 24 min into the reaction using 7.5 ug/mL NTR.

### Fluorescence responses of probe (1) reacted withrat liver microsomes

**Figure 3:**
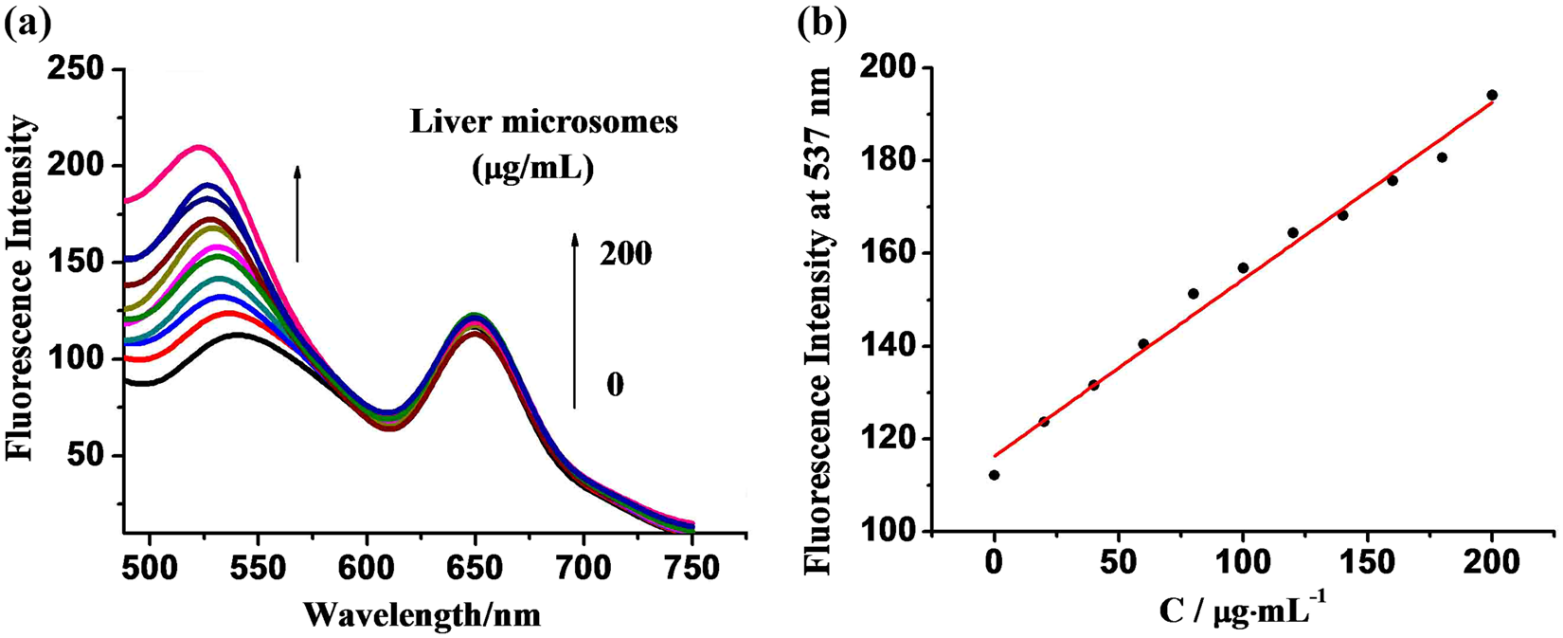
(**a**) Fluorescence emission titration spectra of probe (1) (3.0 × 10^−5^ M) in the presence of varying concentrations of rat liver microsome (0~200 ug/mL) in PBS (0.01 M, pH = 7.4)with 1% DMSO and 80 µM NADH. (**b**) Correlation between emission intensities at 537 nm and concentrations of liver microsome.

Tang *et al*. were the first to use the rat liver microsome as a source of NTR to test the probe’s response^23^. Inspired by their work, we tested the optical spectra of probe (1) towards NTR derived from the rat liver microsome. As shown in Fig. 3a, probe (1) itself showed a weak emission at a wavelength of 650 nm. The addition of rat liver microsome to probe (1) resulted in an additional emission peak at roughly 537 nm. A linear relationin fluorescence intensity was obtained at 537 nm using a NTR concentration in the range of 0–200 ug/mL (Fig. 3).

### The selectivity of probe (1)

**Figure 4:**
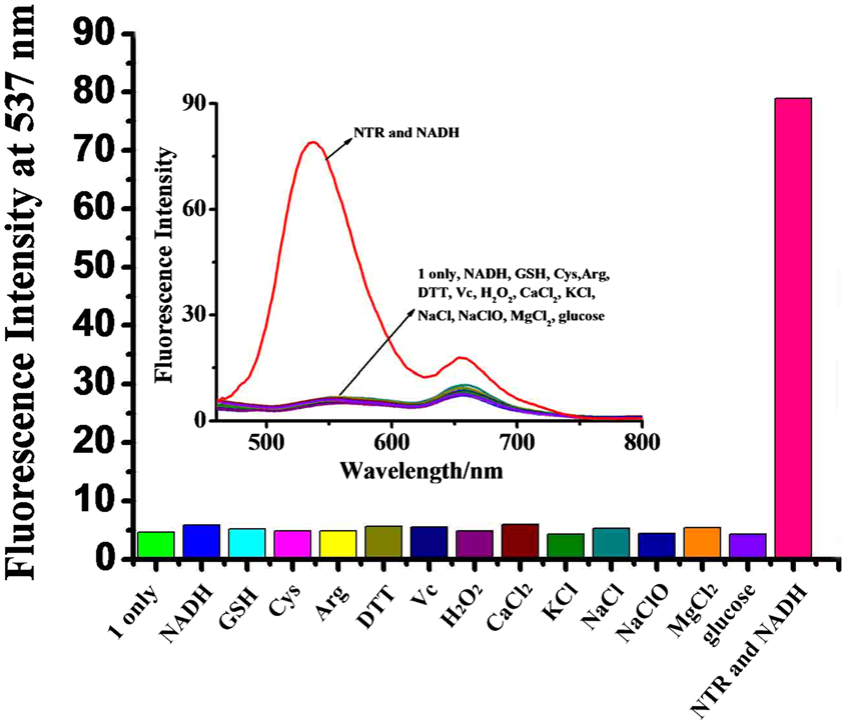
Fluorescence activities of probe (1) (1.0 × 10^−5^ M) to various species: probe (1) only, NADH (10 µM), GSH (1 mM), Cys (1 mM), Arg (1 mM), DTT (1 mM), Vc (1 mM), H_2_O_2_ (1 mM), CaCl_2_ (1 mM), KCl (1 mM), NaCl (1 mM), NaClO (1 mM), MgCl_2_ (1 mM), glucose (1 mM), nitroreductase, and NADH. All measurements were performed in PBS (0.01 M, pH 7.4) with 1% DMSO.

The selectivity of probe (1) was determined in comparison with various analytes, such as glutathione (GSH), cysteine (Cys), dithiothreitol (DTT), arginine (Arg), ascorbic acid (Vc), H_2_O_2_, NaClO, CaCl_2_, KCl, NaCl, MgCl_2_, glucose, NADH, and NTR with NADH (Fig. 4). We demonstrated that thiol-containing substrates, inorganic salts, and other species total above show no interference toward the reaction between probe (1) and NTR, indicating a high selectivity of probe (1) for the detection of NTR in the test condition. The selectivity of NTR detection is provided by the nitrobenzene group in probe (1) (Fig. 1). The mechanism of probe (1) reduction catalyzed by NTR supporting the results of the selectivity tests was confirmed by mass spectrometry analysis of the reaction products (Fig. S6).

### The cytotoxicity of probe (1)

A low cytotoxicity is aguarantee for the probe’s safe applications in *in vitro* and *in vivo* testing. Cytotoxicity of compound1 was determined by Cell Counting Kit-8 (CKK-8) assays in two control cell lines (MA104 and Ins-1) and two cancer cell lines (A549, SKOV). As shown in Fig. S7, the EC50 values of thecontrolcells (MA104 (33.7 μM); Ins-1 (71.04 μM)) are higher compared tothe EC50 values of the cancer cells (A549 (2.7 μM), SKOV (1.18 μM)). Thus, the low cytotoxiccharacteristicsof probe (1) indicated its safe potentialfor *in vivo* measurement of NTR levels.

### *In vivo* imaging of probe (1)

**Figure 5:**
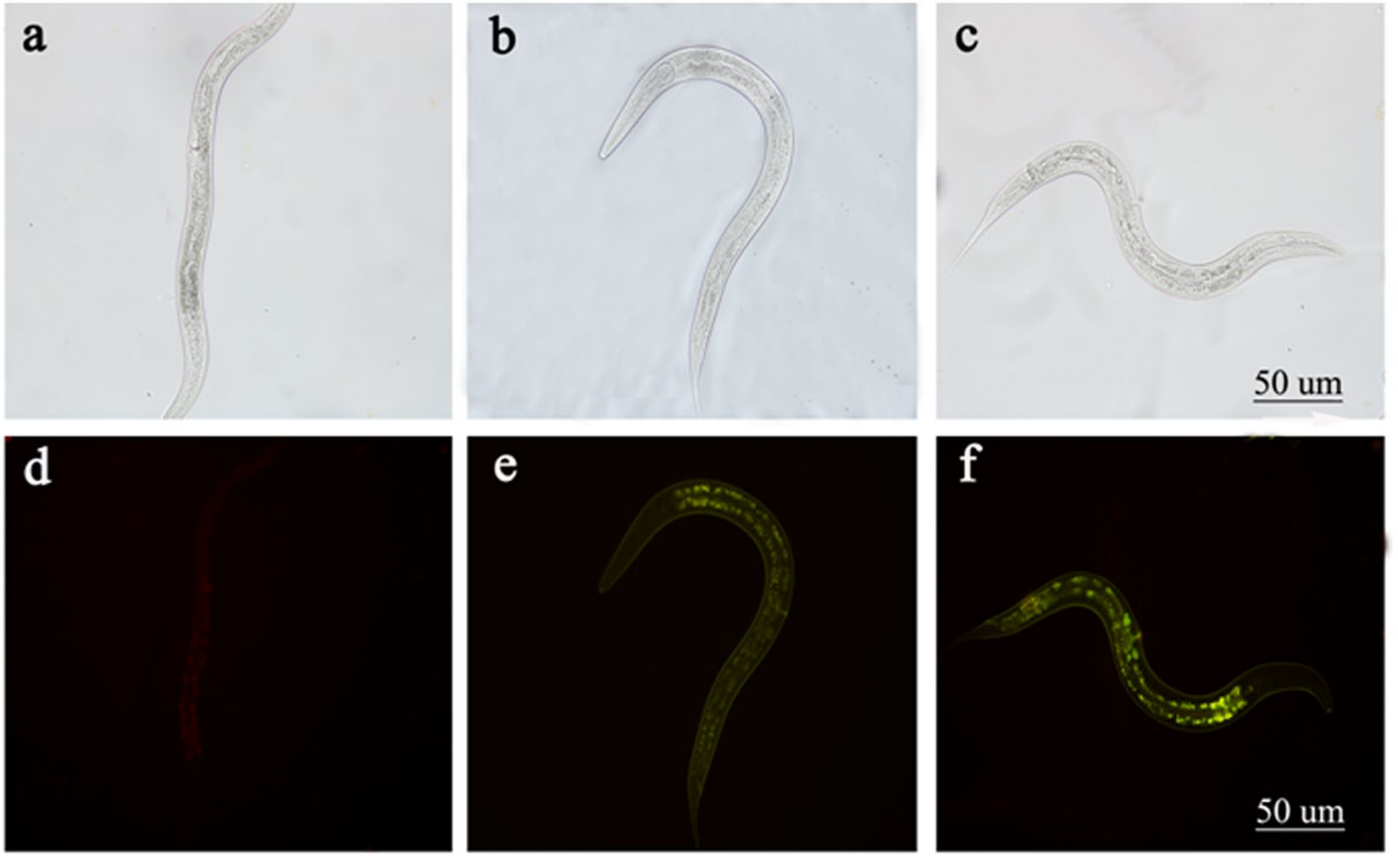
Bright-field image (top) and fluorescent image (bottom) in *C. elegans*. (**a**) Probe (1)(20 µM) only. (**b**) Probe (1) (20 µM), NADH (100 µM), and nitroreductase (NTR) (10 µg/ml). (**c**) Probe1(20 µM), NADH (100 µM), and NTR (20 µg/ml).

The sensitivity of probe 1 was evaluated in *C. elegans* larvae at developmental stage 3 (L3). The larvae were alive and active during exposure to probe (1), indicating the low levels of toxicity of probe (1). To detect exogenous NTR levels in live animals using probe (1), larvae were first incubated in Petri dishes filled with M9 buffer, containing 20 µM 1 for 1 h at 37 °C (Fig. 5a,d). Subsequently, 10 or 20 µg/mL NTR and100 µM NADH was added to the larvaefollowed byincubation for 1 h at 37 °C.

Until addition of NTR (10 µg/ml) and the second substrate (100 µM of NADH), no fluorescence was observed in the pre-treated nematodes. However, addition of 20 µg/ml of NTR, and 100 µM of NADH resulted in a clear bright green fluorescence signal was emitted from the intestinal region, and gonads of larvae, indicating that probe (1) can visualize the detection of exogenous NTR in *C. elegans*.

**Figure 6:**
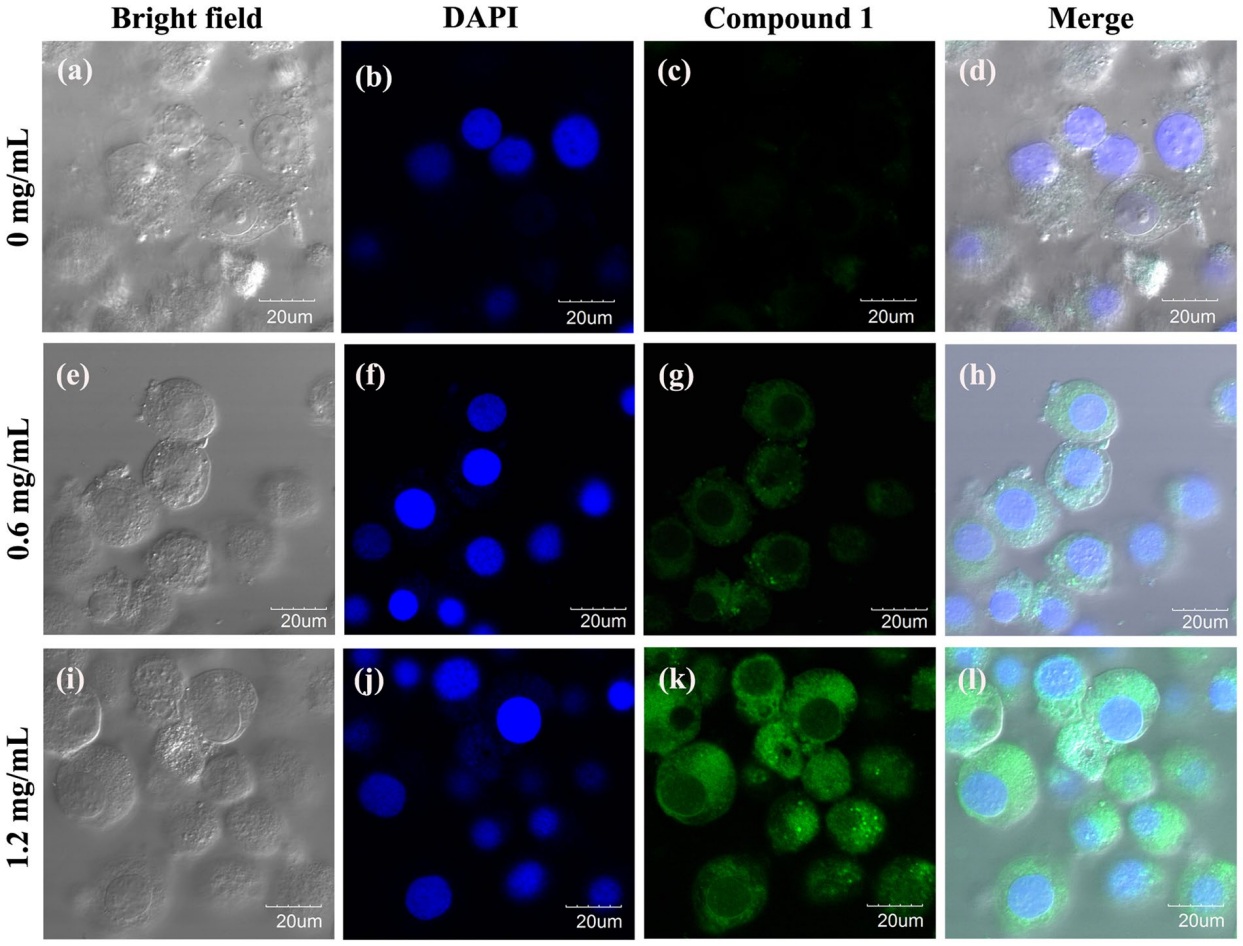
Confocal luminescence images of live Hi5 cells, incubated in PBS for 2 h, at different oxygen levels. (**a**–**d**) Probe (1) only (20 μM); (**e**–**h**) Probe (1) (20 μM), and antioxidant (0.6 mg/mL); (**i**–**l**) Probe (1) (20 μM), and antioxidant (1.2 mg/mL). (**a**,**e**) and (**i**) Are bright-field images. (**b**,**f**) and (**j**) Are blue channels collected at 425–475 nm, stained with DAPI. (**c**,**g**) and (**k**) are green channels collected at 495–550 nm, stained with probe 1. (**d,h** and **l**) Are mergedimages.

Fluorescent imaging of Hi5 cells treated with probe (1) and DAPI, a commercial nuclei dye, was performed to evaluate the potential of probe (1) topenetrate into live cells. Undernormoxic conditions, Hi5 cells were incubated for 2 h at 37 °C with 20 μM of probe (1), then nuclei were stained with 1uM DAPI for 5 min at 37 °C. As shown in Fig. 6, a very weak green fluorescence signal was released from the cytoplasm from cells that were incubated with probe (1). Treatment of the cells with the antioxidant (Glutathione ethyl ester) at different concentrations (0.6 or 1.2 mg/mL) results in a significant decrease in oxygen level.The fluorescence intensity of the probe in the cultured cells increased with the increase of antioxidant concentration. The brightest emissionwe observed at an antioxidant concentration of 1.2 mg/mL. Thus, the fluorescent intensity of probe (1) correlated with the intracellular redox status of Hi5 cells. Our data suggested that our approachmaybe used for the detection of hypoxia-induced NTR expression in tumor cells and tissues.

**Figure 7:**
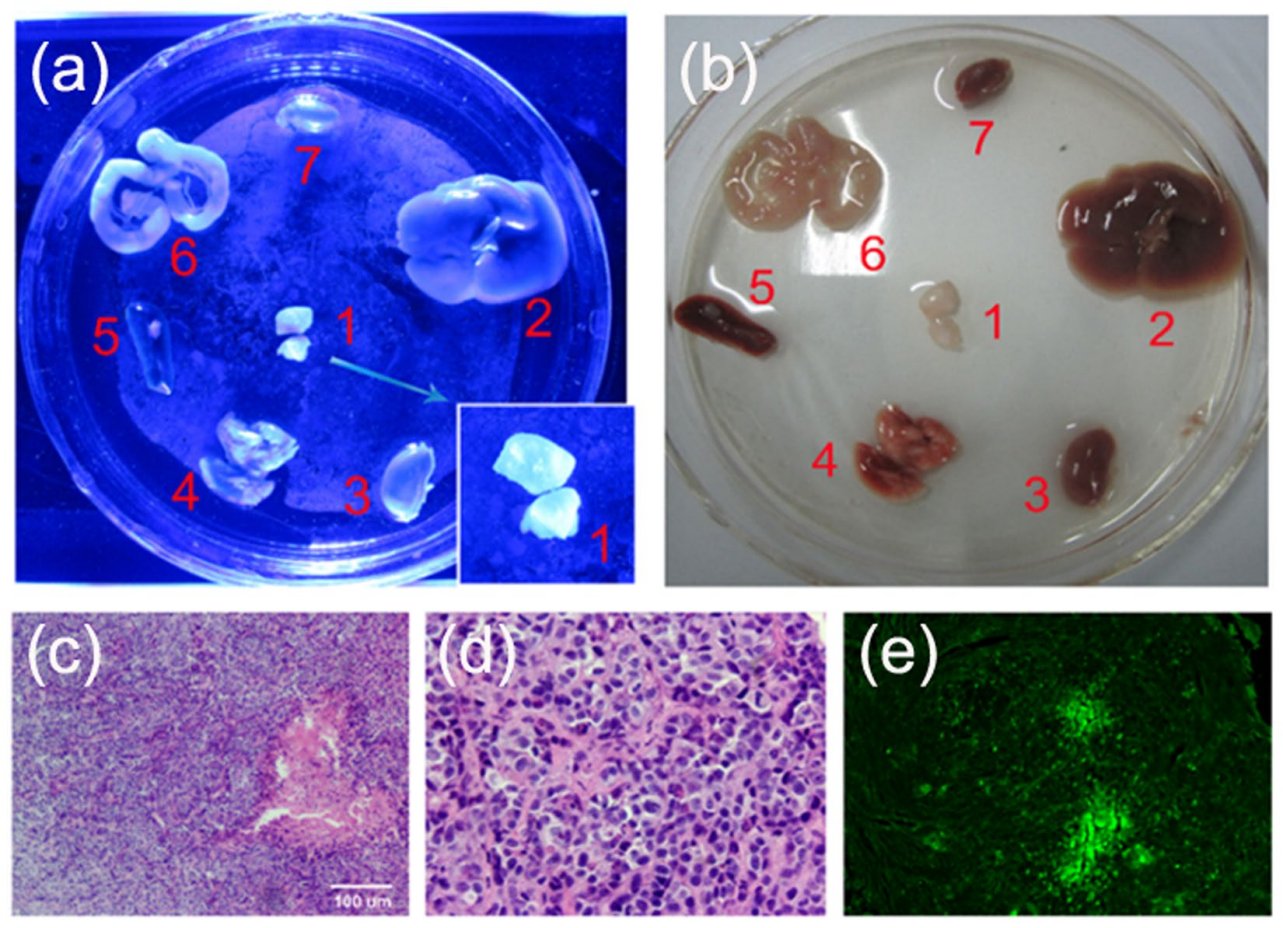
(**a**) and (**b**) Representative images of dissected organs of amouse bearing HEPG-2-induced tumors. The mouse was sacrificed and organs were removed and incubated with 100 μM of 1 for 5 hours under hypoxic conditions. 1. HEPG-2 tumor; 2. lung; 3. liver; 4.kidney; 5. spleen; 6. intestine; 7. heart. (**c**) H&E staining of murine sarcoma HEPG-2-induced tumor (×100). (**d**) H&E staining of murine sarcoma HEPG-2-induced tumor (×400). (**e**) Immunofluorescence of HEPG-2 tumor by Glypican 3 staining.

In solid tumors, the level of hypoxia increased with increasing tumor size and correlated with NTR over-expression. Therefore, we hypothesized that probe (1) with NTR as a targetmay evaluate the hypoxia levels in tumor tissues. To explore the diagnostic potential of our approach, a tumor model was established in which HEPG-2 cell (1 × 10^7^/mL) in 100 μL PBS were subcutaneously injected into the left flank of the mice. When the tumor volume reached 1 cm^3^, mice were sacrificed by cervical vertebra dislocation, and organs including lung, liver, kidney, spleen, intestine, heart and tumors were collected for organ imaging. After 5 h of incubation with 100 μM of probe (1) at 37 °C under hypoxic conditions (5% O_2_), tumor tissue demonstrated a strong fluorescence signal when compared to the surrounding tissues (Fig. 7a). Tumor tissue morphology was confirmed by H&E staining (Fig. 7c,d) Immunofluorescence staining using Glypican-3 as a marker of liver cancer, wasused for tumor identification (Fig. 7e). And showed that probe (1) could successfully be used to characterize hypoxic tumors. Thus, we demonstrated that probe (1) may be applied as a selective and sensitive bioimaging probe in a mouse model of hypoxic tumors.

In summary, we have successfully developed probe (1), a NTR-targeted fluorescent probe that detects hypoxic conditions intumor cells and tissues. Our approachis based on the ability of NTR to catalyze the reduction of probe (1) with outstanding sensitivity and selectivity. Probe (1) was used for detecting exogenous NTR in *C. elegans*, and the hypoxic status in Hi5 cells. Moreover, in mice bearing HEPG-2 induced tumors, we demonstrated that probe (1) is a useful tool for the detection of hypoxic conditions in tumortissue. We anticipate that the application of probe (1) will reveal the bio-roles of NTR, and the relation of NTR and hypoxia in pathological processes.

## Methods

All methods were performed in accordance with the relevant guidelines and regulations.

### Synthesis of probe (1)

(For detailed synthetic procedures, please see the Supporting Information.) A mixture of compound 2 (0.2 g, 0.64 mmol), 1-(bromomenthyl)-4-nitrobenzene (0.166 g, 0.768 mmol), K_2_CO_3_ (0.442 g, 3.2 mmol), and NaI (0.048 g, 0.32 mmol) in 25 mL CH3COCH3 were heated under reflux for 4 h. After completion of the reaction, the mixture was cooled to room temperature, and condensed under reduced pressure. Chromatography of the crude product on silica gel using CH_2_Cl_2_ as an eluent resulted in a pale reddish orange solid product of 258.3 mg (yield = 90%). ^1^H NMR (500 MHz, DMSO-*d6)* δ(ppm): 8.69 (d, 1 H), 8.27-8.26 (d, 2 H), 7.91(d, 1 H), 7.77-7.69 (d, 6 H), 7.59 (d, 1 H), 7.36-7.33 (d, 1 H), 7.13-7.12 (d, 2 H), 6.95 (d, 1 H), 5.34 (s, 2 H). ^13^C NMR (125 MHz, DMSO-d6) δ(ppm):160.18, 158.91, 153.32, 152.41, 147.45, 144.99, 138.85, 135.75, 130.47, 128.67, 126.50, 125.01, 124.02, 119.41, 117.82, 117.65, 117.49, 116.32, 115.86, 106,54, 68.59, 60.03.

### Preparation of the test solution

PBS(pH = 7.4), and analyticalgrade DMSO were used for spectroscopic studies. The probe 1stock solution (1 × 10^−3^ mol/L) was preparedin analyticalgrade DMSO. The concentration of probe (1) in test solution was 1 × 10^−5^ mol/L, and was prepared by mixing 50 μL stock solution and 4.95 mL PBS. Then 10 μL NADH, and increasing concentrations of NTR (0, 2.5, 5, and 7.5 μg/mL) were added to the test solution. For selectivity studies, various analytes, including glutathione (GSH), cysteine (Cys), dithiothreitol (DTT), arginine (Arg), ascorbic acid (Vc), H_2_O_2_, NaClO, CaCl_2_, KCl, NaCl, MgCl_2_, glucose, NADH, and NTR were added to probe (1).

### Cyotoxicity assays

To investigate the cytotoxicity of probe (1), CCK-8 tests were performed in A549 and SKOV cancer cells, and MA104 and Ins-1 control cells. Cells were seeded into 96-well microplates with 90 µL of cell suspension, and cells were incubated for 24 hours at 37 °C under 5% CO_2_. Cells were washed, followed by addition of increasing concentrations (0.1 μM-10 μM) of probe 1. After 24 h, 10 µL of CCK solution was added to each well,followed by incubation for 2–3 hours at 37 °C. Absorbance was read at 450 nm using a microplate reader (Thermo Multiscan Go).

### Cell culture

Hi5 cells were kindly provided by the institute of life sciences, Yunnan University. Cells were maintained in Dulbecco’s modification Eagle’s medium (DMEM, Invitrogen) supplemented with 10% fetal bovine serum (FBS)at 37 °Cand 5% CO_2_.The cells were seeded ina confocal dish, and incubated for 24 h at 37 °C at 5% CO_2_. Then the cells were incubated with 20 μM probe (1), and different concentrations of the antioxidant (0 mg/mL, 0.6 mg/mL, 1.2 mg/mL) for 2 h at 37 °C in 5% CO_2_.

### C. elegans culture

The *C. elegans* wild type strain N_2_ was acquired from the institute of life sciences, Yunnan University. *C*. *elegans* in the larval stage 4 (L4) were incubated at 20 °C for 1 h in Petri dishes filled with M9 buffer, containing probe (1) (20 µM). After incubation, the exposed nematodes were washed three times with M9 buffer andcentrifuged at 3000 r/min for 2 minutes. Then NADH (100 µM), and different concentrations of NTR (0 µg/mL, 10 µg/mL, and 20 µg/mL) were added to the nematode. Images of the mounted nematodes were acquired by a fluorescent microscopy Olympus BX51.

### Co-staining of cells

To confirm that probe (1) specifically stained the cytoplasm where NTR ismainly generated, co-staining studies were performed. Undernormoxicconditions, Hi5 cells were incubated with 20 μM of probe (1) for 2 h at 37 °C. Underhypoxic conditions, antioxidant (Glutathione ethyl ester) at different concentrations (0.6 or 1.2 mg/mL) was addedand nuclei were stained for 5 min with 1 μM DAPI. Emission was analyzedusingblue channels and a wavelength of 425–475 nm for DAPI, and green channels at 495–550 nm for probe (1). Fluorescence imaging was performed usingan Olympus FV1000 confocal microscopy with a 40× objective lens.

### Establishinga human hepatoma tumor-bearing nude mouse model

All procedures involving animals were approved by the Committee on the Use of Live Animals in Teaching and Research of Yunnan Minzu University. Female SPF nude mice (weighing 16–20 g) were randomly divided into two experimental groups. To establish a human hepatoma tumor model, 1 × 10^7^/mL HEPG-2 cells were diluted with 100 μL PBS, and subcutaneously injected into the right armpit of the mice. Weights were measured and general behavior and body condition was assessed. On day 21 after injection, all mice were sacrificed, and tumors were isolated and measured bya Vernier caliper to calculate tumor volume (V) = a × b^2^/2).

### Immunohistochemistry

Tissues were fixed for 48 h in 4% formalin,and dehydrated in a graded series of ethanol. Tissues were embedded in paraffin and sliced into 4 μm sections. Subsequently, sections were deparaffinized, and stained with hematoxylin and eosin (HE) for histological analysis. After dewaxing and dehydration, sections were washed 3 × 5 min with distilled water. Then the sections were boiled in citrate buffer solution for 30 min, followed by washing in PBS for 3 × 5 min. The sections were incubated for 30 min in 10% serum at 37 °C. Serum was removed, and a rabbit-anti mouse monoclonal anti-Glypican 3 antibody (1:1,000, Abcam, MA, USA) was added to each well, and incubated overnight at 4 °C. After rinsing with PBS, sections were incubated with a FITC-conjugated goat-anti rabbit antibody (1:2,000, Abcam).After another incubation for 2 h at 37 °C, the sectionswere washed with PBS for 3 × 5 min.Then sections were examined under a fluorescence microscope (Leica DMI3000B, Leica Microsystems Ltd., Wetzlar, Germany) using a mercury laser to excite FITC at 488 nm. Emissionwas recorded at 525 nm.

The anti-HIF1α antibody (CST, MA, USA; 1:100 dilution) was used for immunohistochemistry (IHC) after validation and optimization. IHC was performed according to published protocols^24^. with a standard diaminobenzidine (DAB) staining protocol. Briefly, antigens were first retrieved by boiling the slides in 0.1 M Tris-HCl (pH 9.0) buffer for 5 min. After washing with 0.1 M PBS containing 0.1% Triton X-100, slides were then incubated with the anti-HIF1α primary antibody at 4 °C overnight. Then the biotinylated anti-mouse secondary antibody and the avidin-biotin complex (Vector Laboratories, Burlingame, USA) were applied to the slides sequentially. Finally, slides were incubated with DAB (Vector Laboratories) until suitable staining developed.

## Electronic supplementary material

Supplementary info
