# Supplementary material for: Hypoxia imaging in cells and tumor tissues using a highly selective fluorescent nitroreductase probe
Source: Sci Rep. 2017 Aug 23;7:9174. doi: 10.1038/s41598-017-09525-2 (PMC5569069; doi:10.1038/s41598-017-09525-2)
Supplement: Supplementary file 1 — Supplementary info [file 41598_2017_9525_MOESM1_ESM.doc]

Supporting Information

Hypoxia imaging in cells and tumor tissues using a highly selective fluorescent nitroreductase probe

Dan Yang ^a^, Hang Yu Tian ^b^, Tie Nan Zang ^a^, Ming Li ^b^, Ying Zhou ^a^,*, and Jun Feng Zhang ^c^,*

a College of Chemical Science and Engineering, Yunnan University, Kunming 650091, China; E-mail: [yingzhou@ynu.edu.cn](mailto:yingzhou@ynu.edu.cn)
b Institute of Life Sciences, Yunnan University, Kunming 650000, China

c College of Chemistry and Chemical Engineering, Yunnan Normal University, Kunming 650500, China; E-mail:junfengzhang78@aliyun.com

**Experimental Section**
**General methods**

Unless otherwise noted, materials were obtained from commercial suppliers and were used without further purification. Flash chromatography was carried out on silica gel (230-400 mesh). ^1^H NMR spectra were recorded using BRUKER DRX 500 spectrometer; ^13^C NMR spectra were recorded using BRUKER DRX 500 spectrometer; mass spectrometry was recorded with Agilent LC/MSD TOF mass spectrometer. The UV-Vis spectra were obtained using UV-240IPC spectrophoto meter. The fluorescence spectra were obtained with F-4500 FL spectrometer with a 1cm standard quartz cell.

**Confocal microscopy imaging**

The images were observed with a fluorescence microscope (Olympus FV1000;Olympus,
Tokyo, Japan) which was equipped with U-MWU2.

**The linear range and detection limit**

The detection limit was calculated based on the method reported in the previous literature [1]. The fluorescence emission spectrum of **1** was measured by twenty times and the standard deviation of blank measurement was achieved. The fluorescence intensity at 537 nm was plotted as a concentration of nitroreductase. The detection limit was calculated by using detection limit 3σ/k: Where σ is the standard deviation of blank measurement, k is the slope between the fluorescence intensity versus nitroreductase concentration.

**Synthesis**

**Figure S1.** The synthesis route of compound 1.

Compound **6** is commercial.

Compounds **5**-**3** were synthesized according to the method of X. J. Peng *et al.*

Compound **2** was synthesized according to the method of W. H. Zhu *et al.*

Synthesis of Compound **1**: A mixture of compound **2** (0.2 g, 0.64 mmol), 1-(bromomenthyl)-4-nitrobenzene (0.166 g, 0.768 mmol), K_2_CO_3_ (0.442 g, 3.2mmol ) and NaI (0.048 g, 0.32 mmol) in 25 mL CH_3_COCH_3_ were heated under reflux for 4 h. After the completion of reaction, the mixture was allowed to cool to room temperature and then condensed under reduced pressure, chromatography of the crude product on silica gel using CH_2_Cl_2_ as eluent to afford a pale reddish orange solid product 258.3 mg (yield = 90%) . ^1^H NMR (500 MHz, DMSO-*d6*) *δ*(ppm): 8.69 (d, 1H), 8.27-8.26 (d, 2H), 7.91(d, 1H), 7.77-7.69 (d, 6H), 7.59 (d, 1H), 7.36-7.33 (d, 1H), 7.13-7.12 (d, 2H), 6.95 (d, 1H), 5.34 (s, 2H). ^3^C NMR (125 MHz, DMSO-*d6*) *δ*(ppm):160.18, 158.91, 153.32, 152.41, 147.45, 144.99, 138.85, 135.75, 130.47, 128.67, 126.50, 125.01, 124.02, 119.41, 117.82, 117.65, 117.49, 116.32, 115.86, 106,54, 68.59, 60.03.


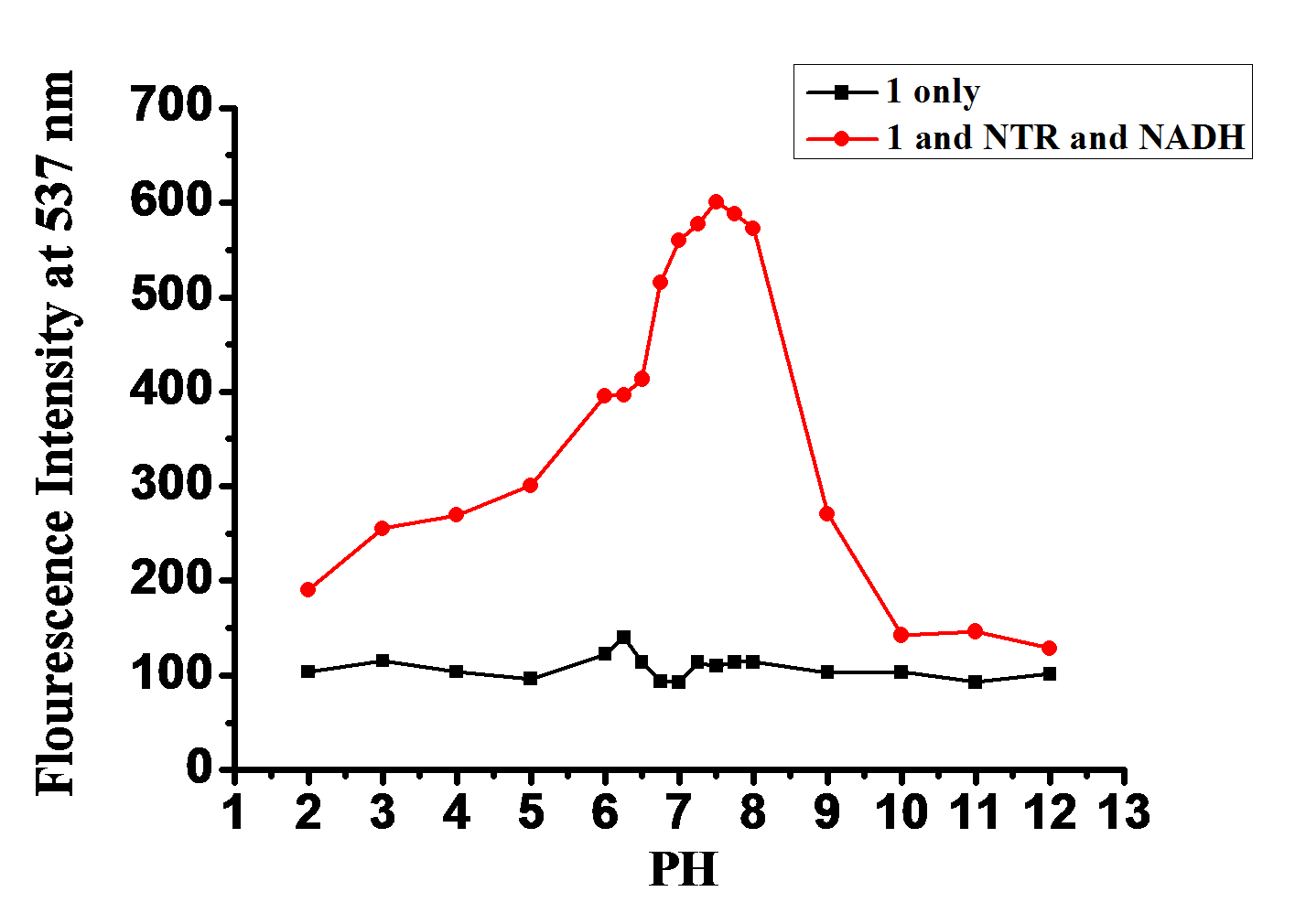


**Figure S2.** Fluorescence responses of 10 μM probe 1 reaction with NTR (7 µg/mL) and NADH (20 μM) in DMSO:PBS (V:V = 1:99) solutions at various pH (2~12).

**
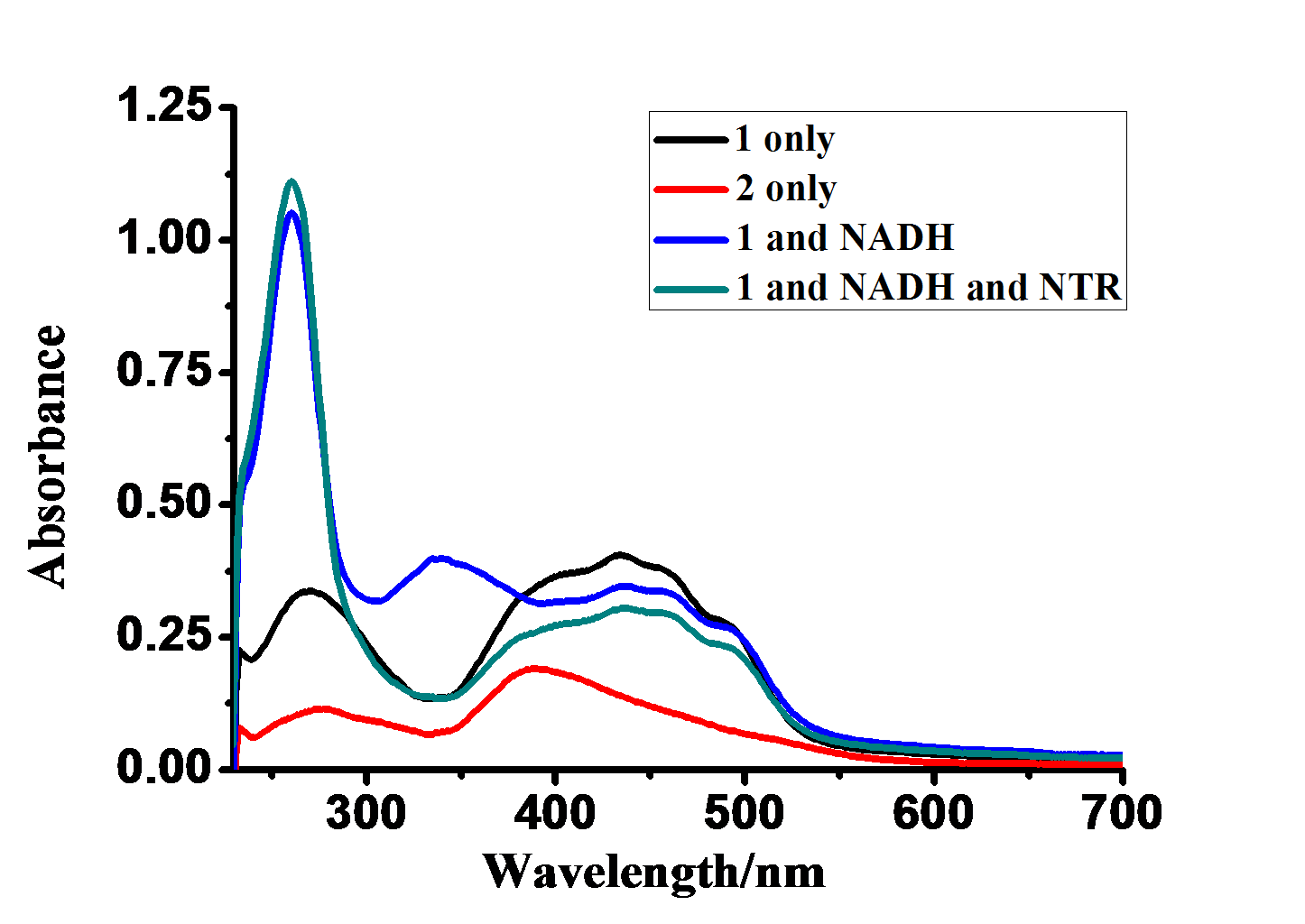
**

**Figure S3.** Absorbance of compound1 (10 μM), compound2 (10 μM), compound1 (10 μM) reaction with NADH (20 μM), compound1 (10 μM) reaction with NADH (20 μM) and nitroredactase (7 μg/mL).


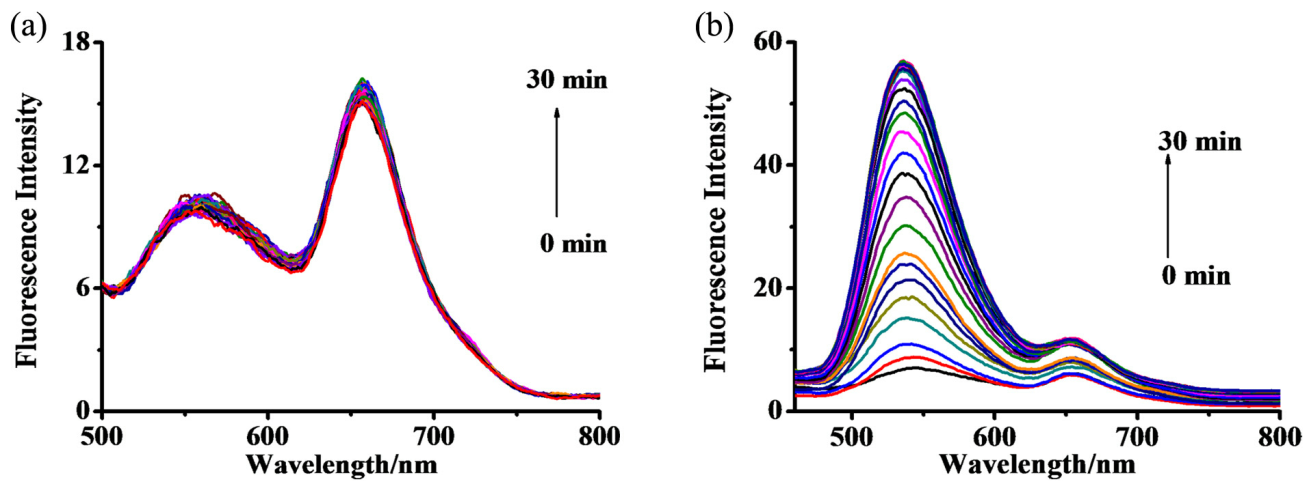


**Figure S4.** Spectra responses of 10 μM probe 1 to 5 μg/mL of NTR in 30 min. The measurements were performed at 37℃ in PBS buffer (0.01 M, pH= 7.4) with 1% DMSO and 20 μM NADH.


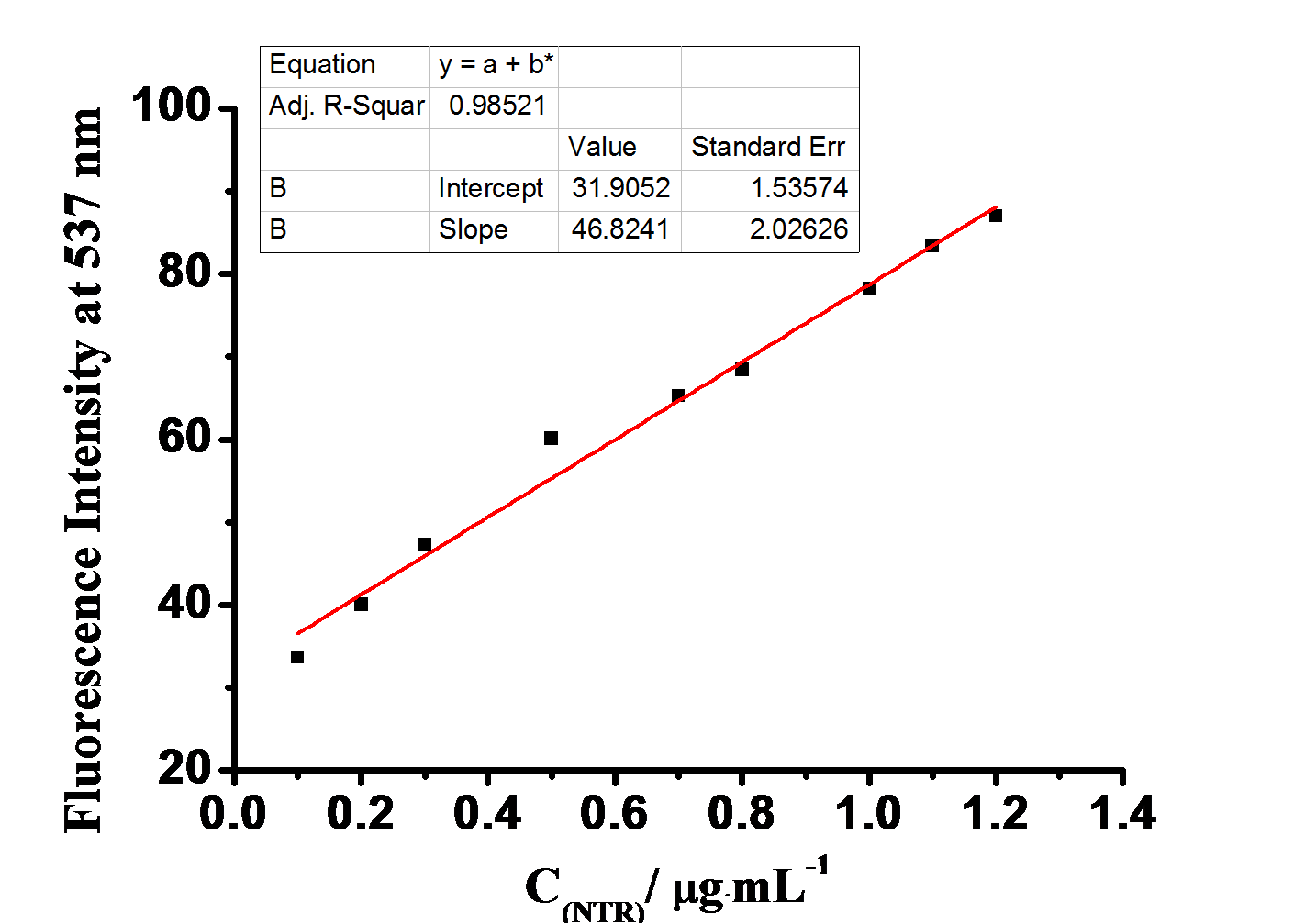


**Figure S5.** Fluorescence intensity of compound1 (2 µM) versus increasing concentrations of nitroreductase.


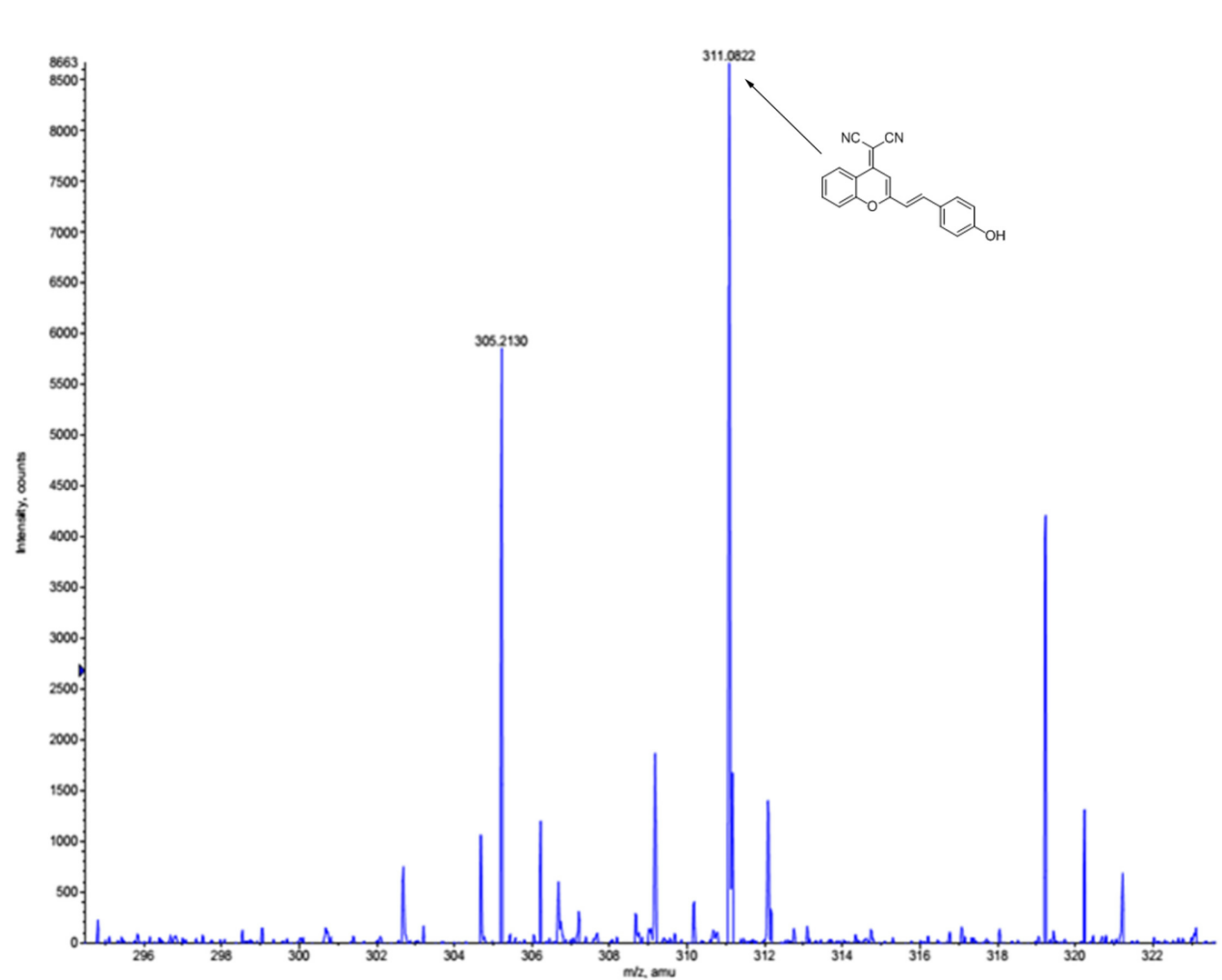


**Figure S6.** The reaction mechanism of compound 1 (up), and TOF-MS of the solution of compound 1 (20 μM) reaction with nitroreductase (15 μg/mL) and NADH (100 μM ) (down).


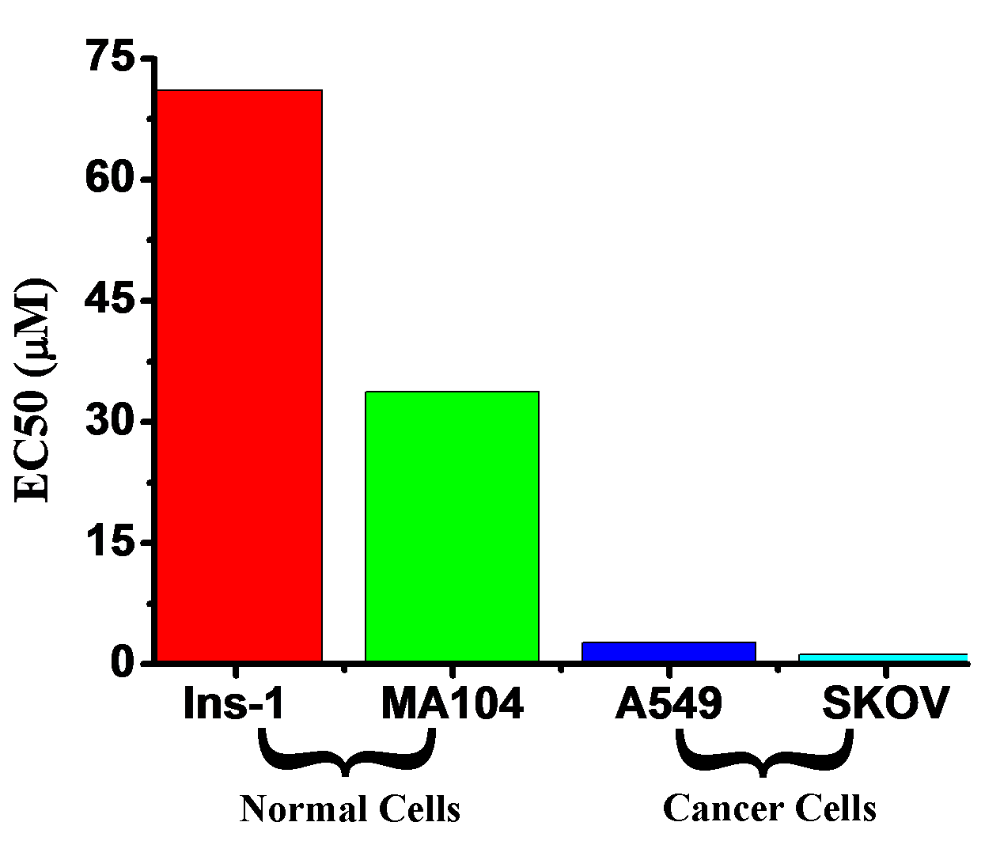


**Figure S7.** EC50 of 1 on Ins-1, MA104, A549 and SKOV cells.

**Figure S8.** ^1^H NMR (DMSO-*d6*, 500 MHz) spectra of compound **1.**

**Figure S9.** ^13^C NMR (DMSO-*d6*, 125 MHz) spectra of compound **1.**

**
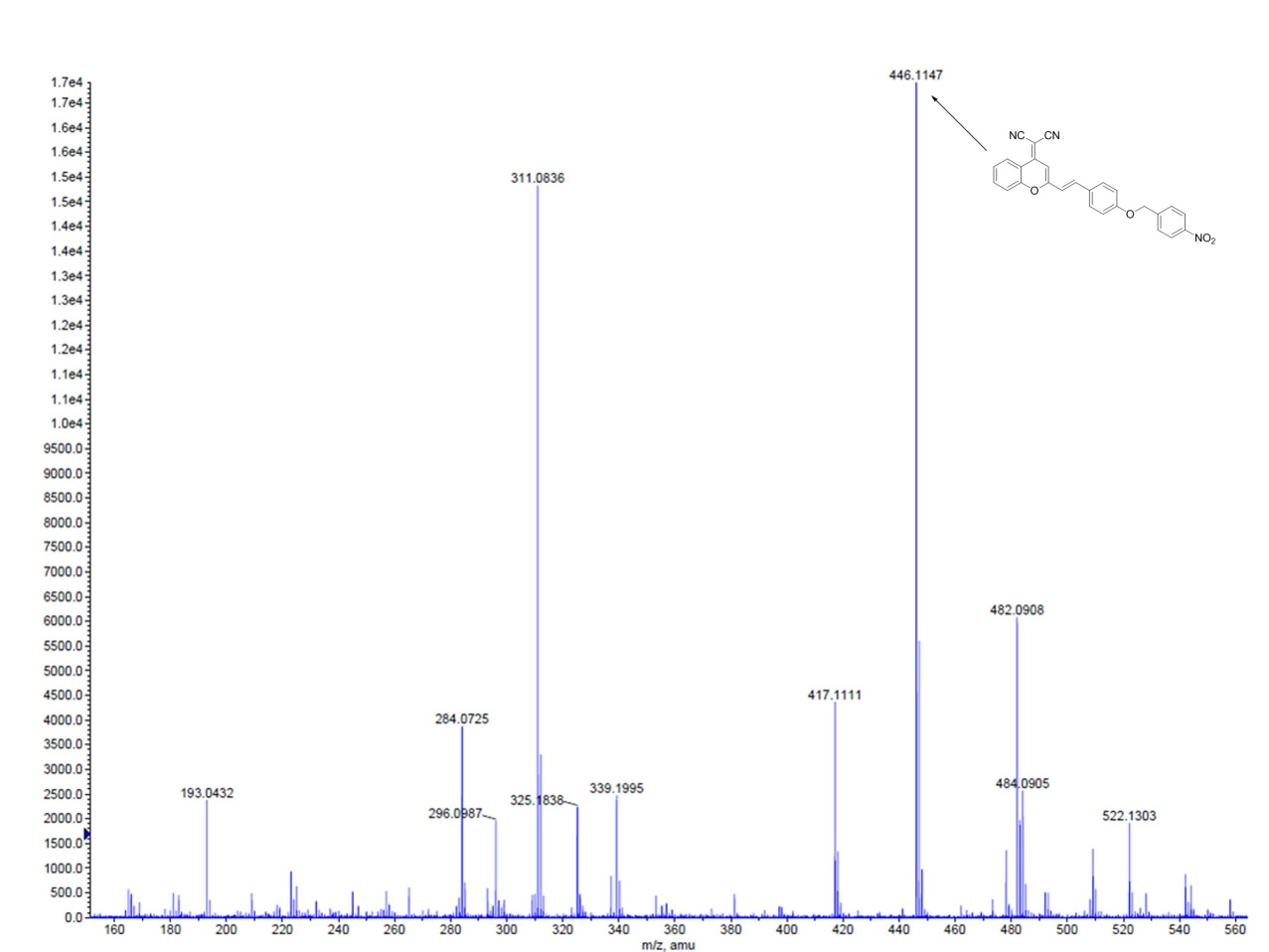
**

**Figure S10.** TOF-MS of the compound1.

**
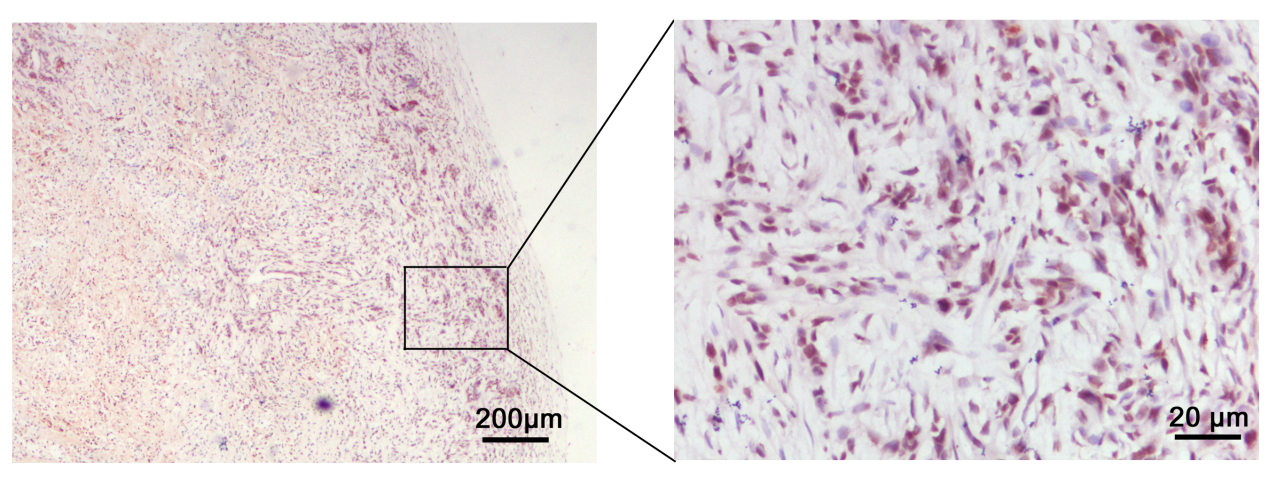
**

**Figure S11.** Cancer xenograft tiusse were fixed and sliced for HIF1α immunohistochemistry staining.
